# Evidence for a Novel Mechanism of the PAK1 Interaction with the Rho-GTPases Cdc42 and Rac

**DOI:** 10.1371/journal.pone.0071495

**Published:** 2013-08-01

**Authors:** Yong Jae Shin, Eun Hye Kim, Adhiraj Roy, Jeong-Ho Kim

**Affiliations:** Department of Biochemistry and Molecular Medicine, The George Washington University Medical Center, Washington, D.C., United of States of America; AMS Biotechnology, United Kingdom

## Abstract

P21-activated kinase 1 (PAK1) is activated by binding to GTP-bound Rho GTPases Cdc42 and Rac via its CRIB domain. Here, we provide evidence that S79 in the CRIB domain of PAK1 is not directly involved in this binding but is crucial for PAK1 activation. S79A mutation reduces the binding affinity of PAK1 for the GTPases and inhibits autophosphorylation and kinase activity of PAK1. Thus, this mutation abrogates the ability of PAK1 to induce changes in cell morphology and motility and to promote malignant transformation of prostate epithelial cells. We also show that growth of the prostate cancer cell line PC3 is inhibited by the treatment of a PAK1-inhibiting peptide comprising 19 amino acids centered on S79, but not by the PAK1 peptide containing the S79A mutation, and that this growth inhibition is correlated with reduced autophosphorylation activity of PAK1. Together, these findings demonstrate a significant role of S79 in PAK1 activation and provide evidence for a novel mechanism of the CRIB-mediated interaction of PAK1 with Cdc42 and Rac.

## Introduction

PAK1 is a major downstream effector of the Rho-GTPases Cdc42 and Rac, which act as molecular switches that transduce various extracellular signals into intracellular responses [1]. PAK1, the best-characterized member of the PAK family, forms a *trans*-inhibited dimer in its inactive state, in which the catalytic domain of one PAK1 monomer is blocked by the autoinhibitory domain (AID) of the other [2], [3]. This autoinhibitory conformation is disrupted by binding of the GTP-bound Cdc42 and Rac to the CRIB (Cdc42/Rac-interactive binding region) domain [4], [5], [6], leading to autophosphorylation at specific sites including T423 within the activation loop and consequent activation of PAK1 [7], [8]. Efficient activation of PAK1 requires its membrane targeting. PAK1 is recruited to the plasma membrane via the SH3-containing proteins Nck and Grb2 [9], [10], [11], where it may be activated by signaling molecules such as PDK1 kinase [12], sphingosine [13], [14] and PIP2 [15] in a manner independent of the GTPases.

PAK1 is frequently overexpressed and hyperactivated by dysregulation of a number of signaling pathways in human cancer cells that are stimulated by growth factor receptors such as EGFR, PDGFR, and VEGFR [16]. The activated PAK1 in turn promotes cancer cell invasion and metastasis by phosphorylating key regulators involved in cytoskeleton reorganization, such as Lim kinase (LIMK) [17], [18] and the P41-ARC subunit of the ARP2/3 [19]. PAK1 activation also stimulates anti-apoptotic pathways, such as the Pak-Raf1-Bad [20], [21] and NFκB [20] pathways, rendering PAK1 attractive as a cancer therapeutic target [22]. There has been a rapid expansion in the development of peptides as potential drugs for cancer therapy over the last decade [23]. HIV-1 TAT protein transduction domain-mediated delivery of macromolecules has emerged as an alternative approach for the internalization of proteins into the cell from the external environment [24]. PAK peptides have been also examined by two groups in different methods; 1) treatment of the PAK peptide (aa 11–23) that interacts with NCK [25]; 2) expression of PAK1 inhibitory domain (aa 83–149) [26].

The crystal structure of the Cdc42-PAK1 complex revealed that the CRIB domain of PAK interacts with Cdc42 by forming an intermolecular β-sheet between residues Y40-I46 of Cdc42 and I76-H83 of PAK but that this interaction seems to be disrupted by the presence of a β-bulge in PAK formed by the sequence _79_SDF_81_ [4]. To get insights into the role of this sequence, here we investigated the effect of the mutation at S79, one of the three residues of the _79_SDF_81_ sequence on the regulation of PAK1 activity. Our biochemical and cell biological studies have demonstrated that S79 plays a crucial role in the PAK1 interaction with Cdc42 and Rac1 and is required for PAK1-mediated malignant transformation of prostate epithelial cells. Thus, this study uncovers a previously unappreciated role of S79 in the regulation of PAK1 activity and demonstrates a novel concept for the activation of PAK1 by the GTPases.

## Results and Discussion

### PAK1^S79^ Plays an Important Role in Autophosphorylation and Kinase Activities of PAK1

**Figure 1:**
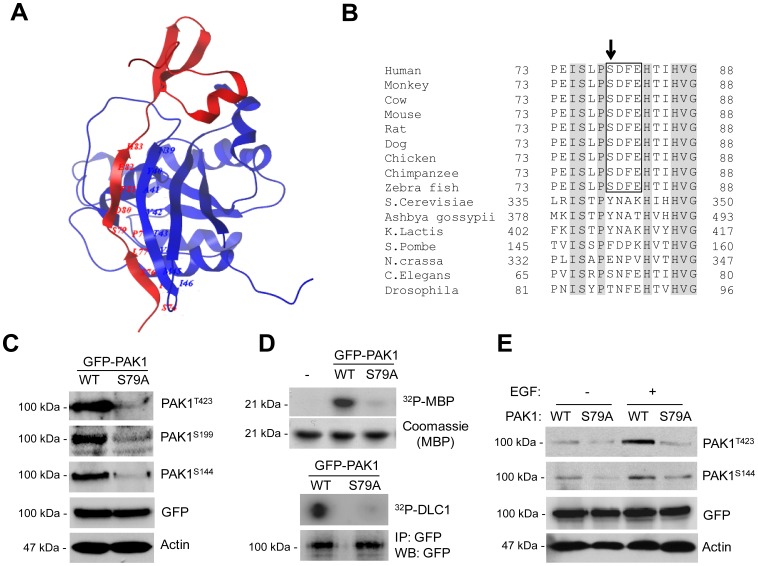
S79 within the CRIB domain is crucial for autophosphorylation and kinase activity of PAK1. Overall Structures of CRIP domain of PAK1 and Sequence Comparisons. (**A**) Ribbon diagram showing a structural overview of CRIP domain of PAK1 (red) with bound Cdc42 (blue). Data Bank ID number 1E0A and visualized using Molsoft ICM Browser (Molsoft, L.L.C., San Diego, CA, USA). (**B**) Multiple sequence alignment of the amino acid sequence of PAK1 from 17 species. (**C**) Western blot analysis of phosphorylation of T423, S199, and S144 and of GFP-PAK1^WT^ or GFP-PAK1^S79A^. (**D**) The kinase activity of PAK1 (GFP-PAK1^WT^ or GFP-PAK1^S79A^) was assessed by *in vitro* phosphorylation assay using MBP (upper) or DLC1 peptide (lower) as substrate. (**E**) Western blot analysis of the effect of S79A mutation on the PAK1 autophosphorylation using anti-T423 and anti-S144 phospho-specific PAK1 antibodies. Cells were unstimulated (−) or stimulated (+) by EGF (100 ng/ml).

PAK1 interacts with Cdc42 and Rac via the CRIB domain (amino acid residues 74-88) [1]. A study indicated that _79_SDF_81_ motif is positioned near the center of the CRIB domain and appears to disrupt the intermolecular β-sheet interaction between residues Y40-I46 of Cdc42 (blue) of Cdc42 and I76-H83 of PAK (red) [4] (Fig. 1A). Our sequence alignment showed that this motif is conserved only in higher eukaryotic organisms, suggesting diverse mechanisms for the regulation of PAK1 activity (Fig. 1B). Increased PAK1 activity is associated with autophosphorylation at specific sites, including S144, S199 and T423 [14]. To address whether S79 of PAK1 (PAK1^S79^) is required for autophosphorylation and kinase activity of PAK1, we assessed phosphorylation states of these residues in PAK1 (WT) and PAK1^S79A^ by IP/Western blot analysis and PAK1 kinase activity by in vitro kinase assay, respectively. Our results show that S79A mutation significantly decreases the phosphorylation of the three residues (Fig. 1C) and kinase activity of PAK1 toward the PAK1 substrates MBP (myelin basic protein) and DLC1 (dynein light chain 1) peptide (Fig. 1D). PAK1 activation is stimulated by a variety of factors including epidermal growth factor (EGF) [16]. We found that S79A mutation markedly decreases EGF-induced PAK1 autophosphorylation at both S144 and T423 (Fig. 1E).

### PAK1^S79^ is Required for the Interaction of PAK1 with Rac1

**Figure 2:**
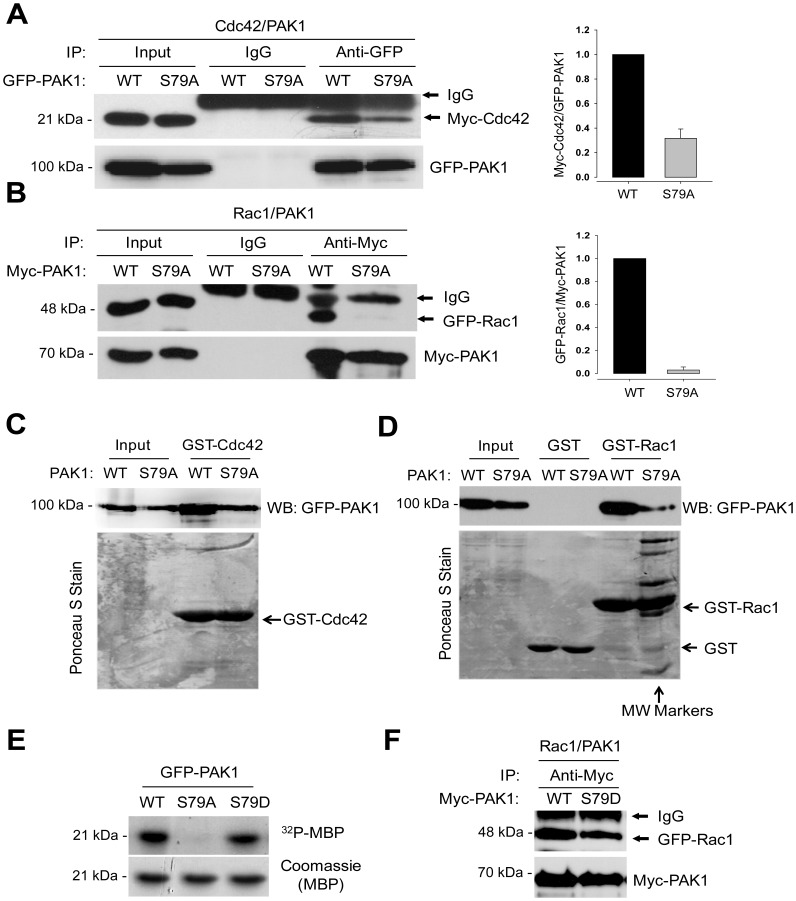
S79A mutation impairs the interaction of PAK1 with Cdc42 and Rac1. (**A**) Interaction between Myc-Cdc42 and GFP-PAK1. GFP-PAK1^WT^ or GFP-PAK1^S79A^ was coexpressed with Myc-Cdc42 in 293T cells. Cell extracts were immunoprecipitated with anti-GFP antibody (IP) and then immunoblotted with anti-GFP or anti-Myc antibody (left). (**B**) Interaction between GFP-Rac1 and Myc-PAK1. Cell extracts were immunoprecipitated with anti-Myc antibody (IP) and then immunoblotted with anti-Myc or anti-GFP antibody. (**C and D**) Cell lysates prepared from 293T cells expressing GFP- PAK1^WT^ or GFP- PAK1^S79A^ were incubated with GST-Cdc42 (C) or GST-Rac1 (D) bound to GST-beads. Upper panels, Western blot analysis of the eluates from the beads using anti-GFP antibody; Lower panels, the blots were stained with Ponceau S Stain. MW markers; molecular weight markers.

Given that PAK1 activation is induced by the binding of the activated GTPase to the CRIB domain [4], [5], [6], we next examined S79A mutation effect on the PAK1 interaction with the Cdc42 and Rac1 GTPases. To this end, GFP-PAK1 and GFP-PAK1^S79A^ were coexpressed with Cdc42 or Rac1 in 293T cells, and their interaction was assessed by Co-IP/Western blot analysis. Wild type PAK1 was shown to interact with Cdc42 (Fig. 2A) and Rac1 (Fig. 2B). However, the ability of PAK1^S79A^ to interact with the GTPases was markedly decreased; the binding affinity of PAK1^S79A^ for Cdc42 was reduced by ∼3-fold (Fig. 2A), whereas the Pak1 interaction with Rac1 was barely detectable (Fig. 2B). GST pull-down analysis also revealed a direct interaction between GFP-PAK1 (WT) and GST-Cdc42 (C) or GST-Rac1 (D) bound to GST-beads, whereas PAK1^S79A^ mutant has reduced affinity for both GTPases, for Rac1 in particular. However, we also found that S79D mutation does not affect in PAK1 activity towards MBP (Fig. 2E) and in the PAK1 interaction with Rac1 (Fig. 2F).

### The S79A Mutation Impairs the Ability of PAK1 to Induce Changes in Cell Morphology and Motility

**Figure 3:**
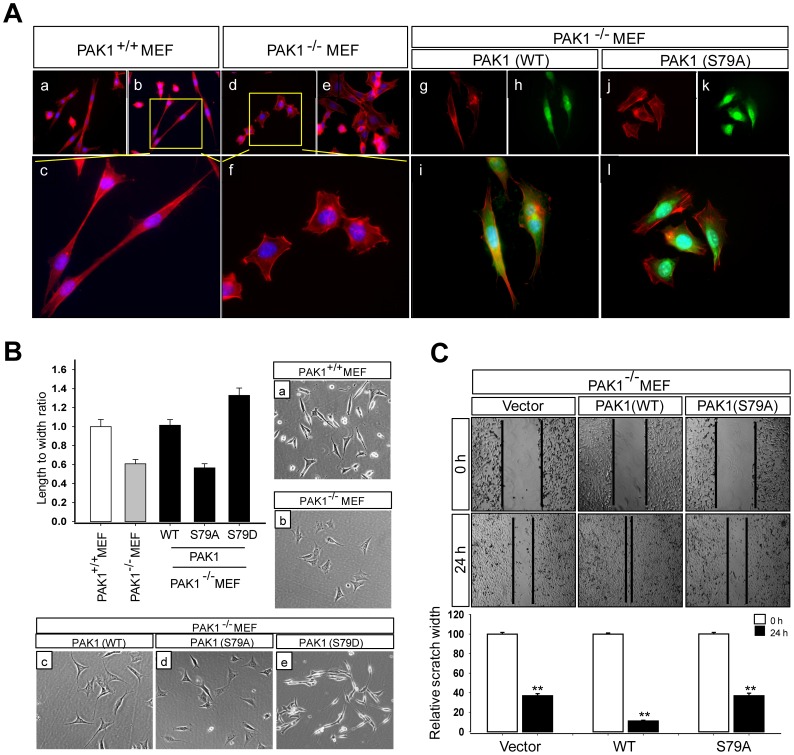
The S79A mutation impairs the ability of PAK1 to regulate cell morphology and motility. (**A**) Wild type (a–c) and PAK1^−/−^ (d–f) MEF cells were stained with the high affinity F-actin probe Phalloidin (red) and DAPI (blue). PAK1^−/−^ MEF cells expressing GFP-PAK1^WT^ (WT, g–i) and GFP-PAK1^S79A^ (S79A, j–l) were stained with Phalloidin (red) and DAPI (blue) and visualized by GFP fluorescence (green). (**B**) Quantification of the length and width (L/W) ratio of MEF cells was obtained as described previously [44]. (**C**) Wound healing migration assays of PAK1^−/−^ MEF cells infected with lentivirus expressing the vector control, GFP- PAK1^WT^, or GFP-PAK1^S79A^. Results were expressed as the percentage of the remaining area determined by normalizing the area of wound after 24 h to the initial wound area at 0 h (set to 100%). Each bar represents the mean ± S.D of five fields measured.

PAK1 is translocated to the focal adhesions and membrane ruffles [27], [28] and the sites of cortical actin remodeling [29] in stimulated cells. We examined the functional importance of PAK1^S79^ by comparing the morphology and motility of PAK1^−/−^ MEF (mouse embryonic fibroblast) cells expressing GFP-PAK1 and GFP-PAK1^S79A^ (Fig. 3A). Wild type MEF cells (PAK1^+/+^) exhibited a bipolar fusiform shape (Fig. 3A, a–c), whereas PAK1^−/−^ MEF cells displayed a more rounded morphology (Fig. 3A, d–f). Expression of GFP-PAK1 in PAK1^−/−^ MEF cells restored the wild type cell shape (Fig. 3A, g–i), whereas GFP-PAK1^S79A^ expression did not rescue this defect (Fig. 3A, j–l). F-actin was colocalized with PAK1, as observed previously in Swiss 3T3 cells [28]; however, this colocalization was significantly reduced in MEF cells expressing PAK1^S79A^ (Fig. 3A and Fig. S1). MEF cells expressing GFP-PAK1^S79A^ exhibited 1.5∼2 fold decrease in the ratio of length to width (L/W), compared with MEF cells expressing GFP-PAK1, whereas those cells expressing GFP-PAK1^S79D^ displayed ∼1.5 fold decrease in the ratio of L to W (Fig. 3B). Wound healing migration assays showed that impaired ability of PAK1^−/−^ MEF cells to migrate into, and close to, the wound was restored by expression of GFP-PAK1 but not of GFP-PAK1^S79A^ (Fig. 3C).

**Figure 4:**
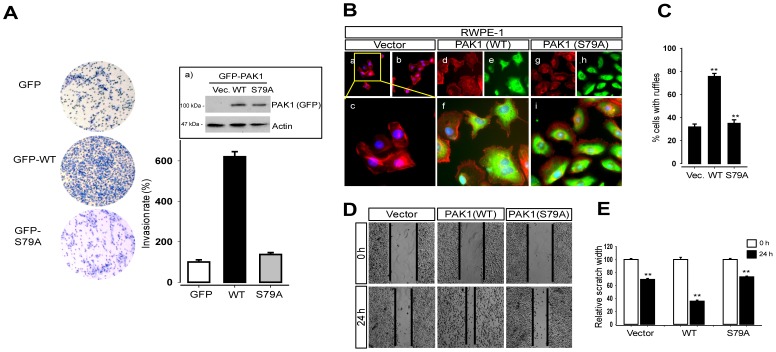
S79A mutation abolishes the migration activity of PAK1. The benign prostate RWPE-1 cells infected with lentivirus expressing the vector plasmid, GFP- PAK1^WT^ (WT), or GFP-PAK1^S79A^ (S79A) were used for experiments (A) to (E). (**A**) The invasion rate was determined by counting the cells that migrated through BME-coated inserts in the Transwell Boyden chamber and expressed as the percentage relative to control (vector). Each bar represents the mean ± S.D of five fields counted. ^a)^ Western blot analysis of PAK1 expression using anti-GFP antibody. Actin was used as an internal control. (**B**) The S79A mutation impairs the ability of PAK1 to regulate formation of membrane ruffles of cells. RWPE-1 cells infected with lentivirus expressing the vector plasmid (a–c), GFP-PAK1^WT^ (WT, d–f) or GFP-PAK1^S79A^ (S79A, g–i) were stained with Phalloidin (red) and DAPI (blue) and visualized by GFP fluorescence (green). (**C**) Quantification of the cells with membrane ruffles (B). (**D**) Wound healing migration assays of RWPE-1 cells expressing GFP-PAK1 or GFP-PAK1^S79A^. (**E**) Each bar represents the mean ± S.D of five fields measured in (**D**).

Next, we compared the ability of wild type and mutant (S79A) PAK1 proteins to confer a migration phenotype on the benign prostate RWPE-1 cells. To this end, RWPE-1 cells were infected with lentivirus expressing the vector plasmid, GFP-PAK1, or GFP-PAK1^S79A^ (S79A). Western blot analysis indicated no significant difference in the expression of GFP-PAK1 and GFP-PAK1^S79A^ in RWPE-1 cells (Fig. 4A ^a)^). Cell invasion assay showed that expression of GFP-PAK1, but not of GFP-PAK1^S79A^, confers an invasive phenotype to RWPE-1 cells (Fig. 4A). We also examined cell morphology of RWPE-1 cells expressing PAK1 or PAK1^S79A^. The formation of membrane ruffles was increased by ∼2.5-fold (Fig. 4C) in RWPE-1 cells expressing GFP-PAK1 (Fig. 4B, d–f), compared with cells expressing GFP-PAK1^S79A^ (Fig. 4B, g–i) or control cells (Fig. 4B, a–c). These results are consistent with previous observations that PAK1 regulates the formation of membrane ruffles of Swiss 3T3 cells [28] and breast cancer cells [30]. We also found that RWPE-1 cells fill ∼30% and ∼60% of the wounded areas by expression of PAK1 and PAK1^S79A^, respectively, at 24 h after scratching (Fig. 4D and Fig. 4E). These observations demonstrate that S79 is crucial for PAK1-mediated cell migration.

### A PAK1-inhibiting Peptide (TAT-PAK1_67–84_) Blocks the Growth of the Prostate Cancer Cell Line PC3

**Figure 5:**
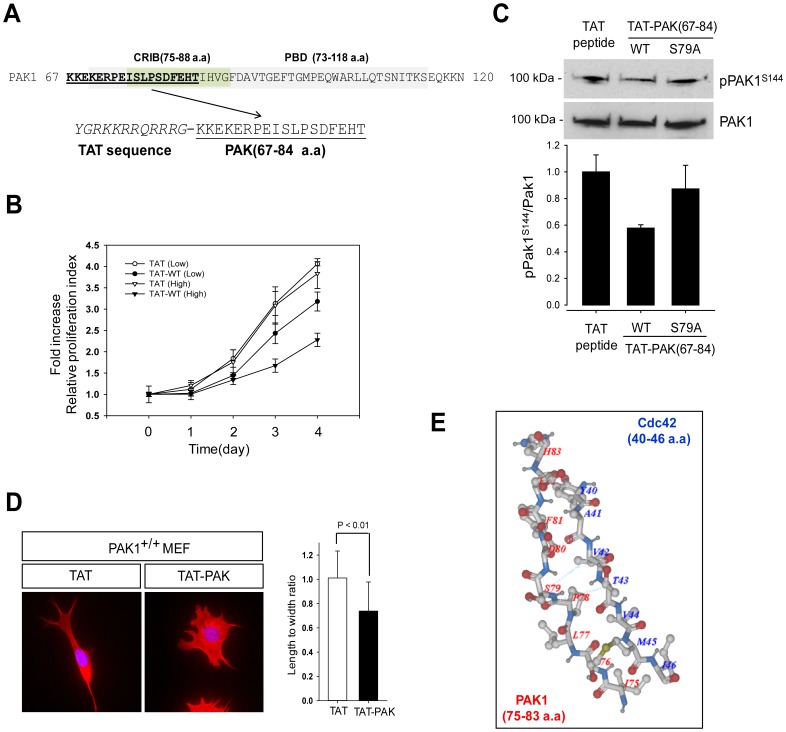
A PAK1-inhibiting peptide blocks the growth of the prostate cancer cell PC3. (**A**) Schematic design of a PAK1-inhibiting peptide (TAT-PAK1 peptide; PAK peptide, aa 67–84). The CRIB domain is underlined. (**B**) PC3 cells were treated with 2 µg/ml or 20 µg/ml of each peptide and examined by MTT assay for 4 days. Cell viability over time was expressed as fold increase, compared with the initial absorbance at day 0. Error bars represent mean ± S.D (n = 5). (**C**) Western blot analysis of S144 phosphorylation of PAK1 in PC3 cells treated with TAT peptide, TAT-PAK1 peptide (WT) or TAT-PAK1 (S79A). (**D**) MEF cells were stained with the high affinity F-actin probe Phalloidin (red) and DAPI (blue) after treatment with with TAT peptide, TAT-PAK1 peptide (WT) or TAT-PAK1 (S79A) (left). Quantification of the length and width (L/W) ratio of MEF cells was obtained as described previously (right). (**E**) A structural overview of CRIP domain of PAK1 (red, 75–83 a.a) with bound Cdc42 (blue, 40–46 a.a). Data Bank ID number 1E0A and visualized using Molsoft ICM Browser (Molsoft, L.L.C., San Diego, CA, USA).

Given that PAK1 activation is triggered by interaction with Cdc42 and Rac1, we examined whether PAK1 activity is reduced by a PAK1-inhibiting peptide. Towards this aim, a peptide containing the CRIB (PAK1_67–84_) was chemically synthesized (Fig. 5A) and fused to the C-terminus of the TAT protein polybasic sequence to facilitate entry into cells as a protein transduction domain [31], [32]. PC3 cells were treated with the peptide at low (2 µg/ml) and high (20 µg/ml) concentrations and were examined for cell growth by MTT assay. We found that treatment of the PAK1 peptide has ∼2-fold inhibitory effect at high dose used (Fig. 5B). To confirm the significance of S79 in the activation process of PAK1 as described above, we also generated a TAT-PAK1_67–84_ peptide with S79A mutation and tested its ability to inhibit PAK1 activation. Western blot analysis indicated that S144 phosphorylation is reduced by ∼40% by the treatment of the PAK1 peptide but is not affected by the treatment of the PAK1 (S79A) peptide (Fig. 5C). We also examined the inhibitory effect of the PAK1-inhibiting peptide on the morphology PAK1^+/+^ MEF cells (Fig. 5D). The bipolar fusiform shape of MEF cells (PAK1^+/+^) was changed to a more rounded morphology by treatment of the PAK1-inhibiting peptide, whereas the treatment of the PAK1 (S79A) peptide has little effect on the morphology of MEF cells (Fig. S2).

### Conclusion

The CRIB proteins such as PAK1-3 kinases [28], ACK tyrosine kinases [33], [34] and the Wiscott-Aldrich-syndrom proteins (WASPs) [35], [36] are activated by direct binding to Cdc42 [37]. While PAK binds to both Cdc42 and Rac, ACK and WASP do not bind to Rac [4]. Structural analyses indicate that the CRIB motif of all the three proteins make an intermolecularβ-sheet interaction with the β2 strand of Cdc42 [4], [38], [39]. Thus, mutations of the amino acid residues that are evolutionarily conserved from human to Drosophila such as I75, S76 and P78 (for sequence alignment, see Fig. 1B) dramatically reduces the binding affinity of PAK for Cdc42 [40]. S79 is conserved only in higher eukaryotes and one of three residues (_79_SDF_81_) forming a β-bulge that disrupts this interaction [4]. The structure of the Cdc42-PAK complex infers that S79 might interact with the V42 of Cdc42 [4] (Fig. 5E), whose mutation does not significantly affect the interaction of Cdc42 with PAK [39]. Hence, S79 is not essential for the PAK interaction with Cdc42. This may be in line with our finding that S79A mutation has little effect on the PAK1-Cdc42 interaction (Fig. 2A–Fig. 2D). However, this mutation is shown to abolish the binding of PAK1 to Rac1, suggesting that PAK1 may bind to Cdc42 and Rac1 by different mechanisms and that S79 may play a key role in enabling PAK1 to distinguish Cdc42 and Rac1. This view is reinforced by the previous work that PAKs bind Rac1 with higher affinity than Cdc42. [41]. The crystal structure of the PAK1-Rac1 complex will help to elucidate the role of S79 in the interaction with and activation by Rac1.

## Materials and Methods

### Reagents and Antibodies

Anti-Pak1, Pak1-pT423, Pak1-pS119 and Pak1-pS144 antibodies were from Cell Signaling. Normal mouse *Protein A/G* PLUS-*Agarose beads*, IgG, anti-actin, anti-c-Myc, and anti-GFP antibodies were from Santa Cruz Biotechnology. Recombinant EGF was from Millipore. Alexa Fluor 568 conjugated-phalloidin was from Invitrogen.

### Cell Culture

The RWPE-1 cells were grown in keratinocyte serum-free medium (K-SFM) containing bovine pituitary extract and epidermal growth factor, as described previously [42]. MEF, PC3, and 293T cells were cultured in RPMI 1640 or DMEM containing 10% FBS and penicillin/streptomycin at 37°C in a humidified atmosphere of 5% CO_2_. PAK1 wild type and PAK1^−/−^ murine embryonic fibroblasts (MEFs) were kindly provided by Dr. Rakesh Kumar [43]. MEFs were isolated from day 13.5 wild-type or Pak1^−/−^ embryos. Wild-type and Pak1^−/−^ MEFs were immortalized with SV40 T antigen and were maintained in DME supplemented with 12% FBS. RWPE-1, PC3, and 293T cells cell lines used in this study were obtained from the American Type Culture Collection (ATCC; Rockville, MD).

### Lentiviral Infection

Lentiviral expression vectors for wild-type GFP-PAK1^WT^ and mutant GFP-PAK1^S79A^ were constructed by subcloning corresponding cDNAs into pLV-puro lentiviral vector, as described previously [42]. For viral production, 293T cells were co-transfected with pLV-GFP, pLV- GFP-PAK1^WT^, or pLV- GFP-PAK1^S79A^, and packaging plasmids (psPAX2 and pCMV-VSV-G) using CalPhos Mammalian Transfection Kit (Clontech).

### Co-immunoprecipitation (IP) and Western Blot Analysis

Cells expressing Pak1, Cdc42, or Rac1 were lysed in lysis buffer (50 mM HEPES, pH 7.4, 150 mM NaCl, 1 mM EDTA, 10% glycerol, 1% Triton X100) containing phosphatase inhibitors (10 mM Na-pyrophosphate, 200 µM Na-orthovanadate, 50 mM Na-flouride). The cell lysates were incubated with appropriate antibodies overnight at 4°C. The bound proteins were eluted by boiling the beads in sodium dodecyl sulfate (SDS) sample buffer for 5 min and were resolved in SDS-polyacrylamide gels. Western blot analysis was performed, as described previously [42].

### Cell Invasion and Migration Assays

Cell invasion assay was performed using the cell invasion kit (Transwell Boyden’s chamber with Transwell® Permeable Support Inserts Coated with Cultrex® BME (basement membrane extract) Corning Costar) according to the manufacture’s instruction. For the cell migration assay, confluent RWPE-1 or MEF cells were scratched with a P-200 pipette tip to cause wounding and subjected to the wound healing assay as described previously [42].

### MTT Cell Proliferation Assays

For MTT cell proliferation assay, PC3 cells were cultured in 96-well microplate. Cell growth was evaluated by replacing the culture media with 200 µl of 0.5 mg/ml MTT-media solution after incubation for 1–4 days. The absorbance was determined at 595 nm using a microplate reader (Bio-Rad Laboratories, iMark).

In all experiments, statistical significance was defined by P-values: *P<0.05, **p<0.005, ***p<0.001 (as compared with control).

## Supporting Information

Figure S1 **PAK1 S79A mutation impairs the ability of Pak1 to change cell morphology.** PAK1^−/−^ MEF cells expressing GFP-PAK1^WT^ (WT) and GFP-PAK1^S79A^ (S79A) were stained with Phalloidin (red) and DAPI (blue) or visualized by GFP fluorescence (green).
(TIF)

Figure S2 MEF cells were stained with the high affinity F-actin probe Phalloidin (red) after treatment with TAT (a–f) or TAT-PAK1 peptide (g–l) [43].
(TIF)
