# Supplementary figures and images for: Evidence for a Novel Mechanism of the PAK1 Interaction with the Rho-GTPases Cdc42 and Rac
Source: PLoS One. 2013 Aug 1;8(8):e71495. doi: 10.1371/journal.pone.0071495 (PMC3731272; doi:10.1371/journal.pone.0071495)

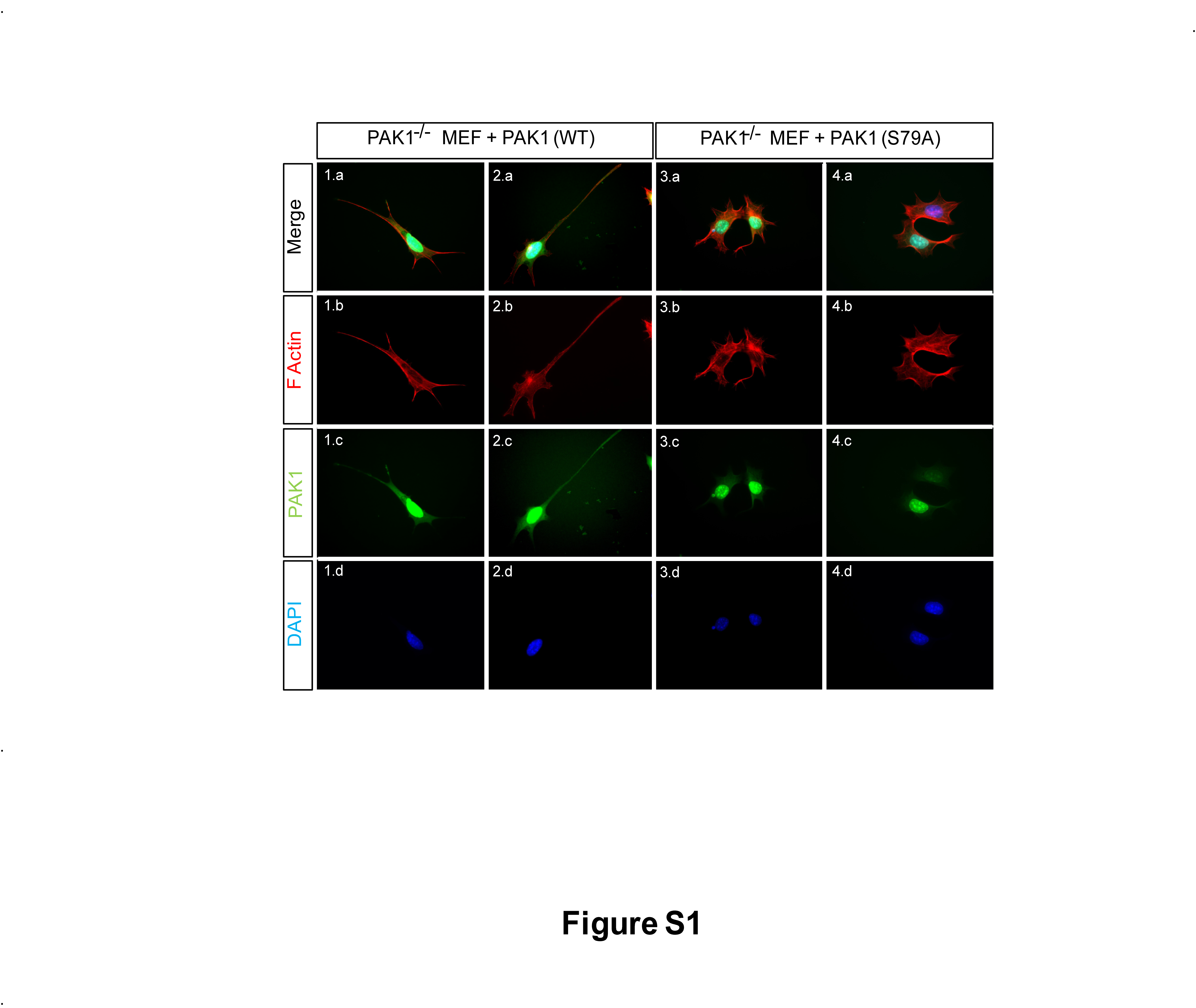

Supplement: Figure S1 — PAK1 S79A mutation impairs the ability of Pak1 to change cell morphology. PAK1−/− MEF cells expressing GFP-PAK1WT (WT) and GFP-PAK1S79A (S79A) were stained with Phalloidin (red) and DAPI (blue) or visualized by GFP fluorescence (green). (TIF) [file pone.0071495.s001.tif]

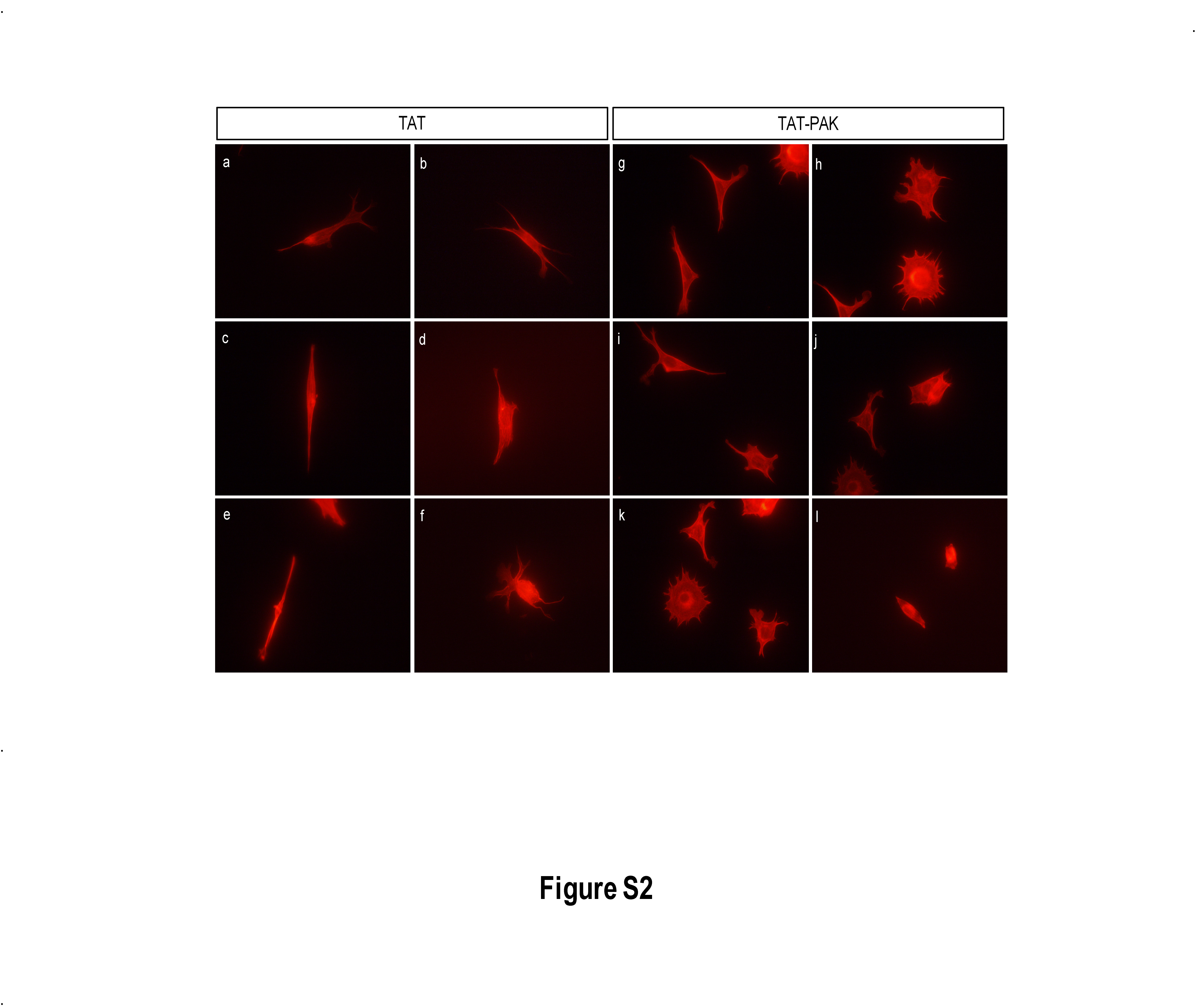

Supplement: Figure S2 — MEF cells were stained with the high affinity F-actin probe Phalloidin (red) after treatment with TAT (a–f) or TAT-PAK1 peptide (g–l) [43]. (TIF) [file pone.0071495.s002.tif]
